# Single-Cell Elasticity Measurement with an Optically Actuated Microrobot

**DOI:** 10.3390/mi11090882

**Published:** 2020-09-22

**Authors:** István Grexa, Tamás Fekete, Judit Molnár, Kinga Molnár, Gaszton Vizsnyiczai, Pál Ormos, Lóránd Kelemen

**Affiliations:** 1 Biological Research Centre, Temesvári krt. 62, 6726 Szeged, Hungary; grexa.istvan@brc.hu (I.G.); fekete.tamas@brc.hu (T.F.); molnartiduj@gmail.com (J.M.); molnar.kinga@brc.hu (K.M.); gaszton@brc.hu (G.V.); ormos.pal@brc.hu (P.O.); 2 Doctoral School of Interdisciplinary Medicine, University of Szeged, Korányi fasor 10, 6720 Szeged, Hungary; 3 Doctoral School of Multidisciplinary Medicine, Dóm tér 9, Hungary University of Szeged, 6720 Szeged, Hungary; 4 Doctoral School of Theoretical Medicine, University of Szeged, Korányi fasor 10, 6720 Szeged, Hungary

**Keywords:** cell elasticity, endothelial cells, optical micromanipulation, holographic optical tweezers, two-photon polymerization, image processing

## Abstract

A cell elasticity measurement method is introduced that uses polymer microtools actuated by holographic optical tweezers. The microtools were prepared with two-photon polymerization. Their shape enables the approach of the cells in any lateral direction. In the presented case, endothelial cells grown on vertical polymer walls were probed by the tools in a lateral direction. The use of specially shaped microtools prevents the target cells from photodamage that may arise during optical trapping. The position of the tools was recorded simply with video microscopy and analyzed with image processing methods. We critically compare the resulting Young’s modulus values to those in the literature obtained by other methods. The application of optical tweezers extends the force range available for cell indentations measurements down to the fN regime. Our approach demonstrates a feasible alternative to the usual vertical indentation experiments.

## 1. Introduction

Autonomous microrobots and microactuators have gained attention recently due to their ability to perform complex tasks on biological targets inside microfluidic environments (channels, reservoirs) without the administration of external physical tools. The targets of these manipulations include protein [1], DNA [2], their association [3] or single cells [4,5]. Furthermore, microtools have been developed to control the flow of the solvent that carries these biological objects [6,7] or to characterize their composition [8]. The complexity of microrobots spans from simple microspheres [1,9] to complex tailor-made microstructures [4,10,11,12], and sometimes a group of such structures is needed to perform specific tasks [13,14]. Most often, these microtools are actuated and guided by optical means, but magnetic [15,16] or acoustic [17] controls are also applied.

Since the size of these microrobots can range from sub-micrometers to a few hundreds of micrometers, they can be easily optimized for the manipulation of single cells. A broad range of tasks can be performed: cells can be actuated with the tools, which includes their simple translation or rotation either on a hard surface [4,5] or in 3D [11,18]; the tools can enhance imaging of cells [12]; the internal structure of the cells can be altered by punching holes in them with the tools [19]; and such microtools have a great potential even in performing cell-to-cell interaction experiments with high precision and selectivity.

In this work, we report on a method that uses tailor-made microtools for the mechanical characterization of single cells. We use optically actuated microtools to make nano-indentations on the cell surface, thereby determining its elastic properties. In order to measure cell membrane elasticity, one needs to realize a small indentation on it with a known radius of curvature of the indenter and a known force; the Young’s modulus then can be calculated from the measured indentation and these parameters [20,21]. In the literature, there are many works reporting on the viscoelastic properties of cells measured by atomic force microscopy (AFM). While AFM can perform this task using forces typically higher than 10 pN, the great benefit of optical manipulation is that the achievable forces complete the range of AFM reaching down to even a few tenths of a pN. Optical tweezers have been applied successfully earlier to measure cells’ Young’s modulus by trapping microbeads of various diameters and pushing them against the cells in an axial direction [20,22,23,24]. These cell indentation experiments use optical forces of less than 10 pN combined with a larger contact surface radius than a typical AFM tip (*r* ≈ 1 μm vs. *r* ≈ 10 nm), which allows only small indentations, and consequently only smaller Young’s moduli can be measured. The smaller force and larger radius of the indenter enable the optical trap-based methods to give a more precise evaluation of the elasticity of softer cells. It is an additional aspect that in the case of the large indentations of AFM, especially if it is coupled with high indentation rates, not only elasticity but also viscosity contributes to the results [20].

On the other hand, in the arrangement where the movement of the optically actuated bead is perpendicular to the surface supporting the cells and parallel to the optical axis, the measurement of bead position is somewhat less accurate. Further, in these situations, the trapping beam illuminates the bead through the cells themselves with such high intensity that it may pose a risk of photodamage on them [25,26,27]. Our approach aims to overcome these drawbacks: the microtool is pressed to the cell in a lateral direction, i.e., perpendicular to the optical axis and to the cell surface that allows for measuring its position and therefore the indentations more precisely, and due to the extended shape of the tool, the trapping foci are micrometers away from the living cells under study posing no risk to them. Our microtool has two functional parts: one that interacts with the optical trapping beams and one that consists of the probe that creates the indentation on the cell surface. Its optical actuation was achieved with holographic optical tweezers (HOT) able to move the structure with 6 degrees of freedom (translations and rotations) with a precision of a few tens of nanometers. The tool can be transported anywhere in a microchannel environment and its tip can be oriented towards any lateral direction so the direction of attack can be freely selected. The probe part in the presented experiments was a tip with a few hundreds of nanometers radius, but the fabrication method allows one to freely change the radius above this value. We performed the indentation experiment on adherent endothelial cells that were cultured on a hard vertical surface that is parallel to the optical axis and formed a confluent layer. Our results demonstrate that the microtool-based method provides a Young’s modulus that fits in the range reported in the literature on this cell type.

## 2. Materials and Methods

### 2.1. Microtool Design and Fabrication

**Figure 1:**
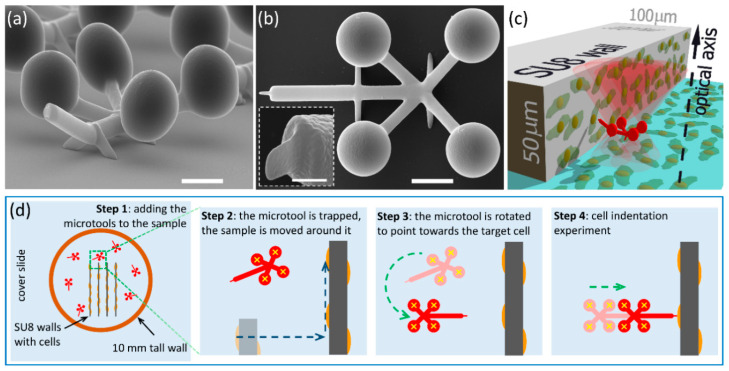
The polymerized microtool used for cell indentation experiments and the sample arrangement. Scanning electron microscopic images of the microtools: (**a**) side view and (**b**) top view (scale bars: 5 μm). It is visible that the tip together with the trapping spheres are at a different plane to the rods connecting them. The insert in (**b**) shows the side view of the microtool’s tip (scale bar: 1 μm). (**c**) 3D schematic view of the experimental arrangement: cells are grown on a vertical wall polymerized from SU8 as well as on the glass substrate forming a confluent layer; the microtool (red structure) that is trapped and actuated with the optical tweezers (red cones) is approaching the cells on the wall with a translation that is perpendicular to the optical axis of the system. Panel (**d**) illustrates the sample assembly process with the microtools (red structures) after being pipetted into the sample well (Step 1) and their alignment towards the target cell (for details see Section 2.4); yellow crosses mark the trap beam positions, dashed blue arrows indicate sample stage movements (Step 2) and dashed green arrows the optical trap actuations (Steps 3 and 4).

The microtools, shown in Figure 1, have two functional parts. The first is used to interact with the optical field and consists of four spheres, arranged at the corners of a square with a side length of 14 μm; these spheres are to be trapped with the HOT. The second is the probe part that creates the indentation on the cells surface. This part is a rod of 2 μm length, created in the plane of the spheres 14 μm away from them; we minimized the diameter of its apex for maximal sensitivity. The rods connecting the spheres and the tip formed an X-shape to minimize interference with the optical field and were slightly offset from the plane of the spheres and the tip. This offset ensured that in the recordings of the experiments these rods were out of focus, and therefore did not add extra features to the image processing when determining the precise position of the structure.

The microtools were made of the photoresist SU-8 (formulation 2007) purchased from Micro Resist Technology GmbH (Berlin, Germany) together with the SU-8 developer (mr-Dev 600, Micro Resist Technology GmbH, Berlin, Germany). Their microfabrication was performed with two-photon polymerization (TPP) with the system described elsewhere [28]. Shortly, the beam of an ultrashort-pulsed laser (C-Fiber A, Menlo Systems GmbH, Martinsried, Germany, *λ* = 795 μm, 100 fs pulse length, 100 MHz repetition rate) was focused into a 20 μm thick photoresist layer supported by a microscope cover slide (type #1, 24 mm × 40 mm, Menzel-Glaser, TS Labor Kft, Budapest, Hungary); the focusing objective was a 100X Zeiss Achroplan, oil immersion (NA 1.25, Carl Zeiss Technika Kft, Budaörs, Hungary). The 3D scanning of the focus within the photoresist layer was carried out by a piezo stage (P-124 731.8L and P-721.10, Physik Instrumente GmbH, Karlsruhe, Germany). The illuminated SU-8 layers were processed with the standard protocol: post-exposure bake carried out at 95 °C for 10 min, development in mr-Dev 600 for 5 min 3 times, rinsing in ethanol for 5 min 3 times and finally drying with a stream of nitrogen. The microtools were removed from their support before the experiment by mechanical means in the aqueous solution of 0.5 m/m% bovine serum albumin; they were then pipetted together with this liquid and transferred to the sample containing the cells.

### 2.2. Cell Culturing

The cells were grown on vertical polymer surfaces (walls), which were parallel to the optical axis, as shown in Figure 1c. The walls were polymerized into SU-8 layers of about 50 μm thickness, supported by cover slides (type #1, 24 mm × 40 mm, Menzel-Glaser) using UV mask lithography. The UV light source was the 365 nm line of a mercury lamp (flood exposure source, model 97435, Newport, Irvine, CA, USA, dose: 340 mJ/cm^2^). The such-created walls were ~5 mm long and 100 μm wide, positioned at the center of the cover slides. A glass ring of 10 mm height was mounted around the walls using Norland optical adhesive, thereby creating a well for cell culturing. The hCMEC/D3 human microvascular cerebral endothelial cells were grown in this well, which was tilted 45 degrees to promote cell adhesion on the vertical parts of the walls. The cells were cultured in EBM-2 medium (Lonza, Switzerland) supplemented with EGM-2 Bulletkit (Lonza, Basel, Switzerland) and 2.5% fetal bovine serum (Sigma, St. Louis, MO, USA) for 3 days before the indentation experiments. The structures, removed from their support, were pipetted in between these walls into the cell culture medium together with about 5 μL liquid that did not alter the composition of the growth medium significantly. The focused beams (red cones in Figure 1c) for the optical trapping passed into the well through its cover slide support.

### 2.3. HOT Setup

The cell stiffness was measured with the tailor-made microtools described above. The microtools were actuated with a holographic optical trap (HOT) system that can create multiple trapping foci and move them in 3D with high precision, as demonstrated in [12]. In the present experiments, we created 4 trapping foci forming a square of 14 μm side length and moved them with their mutual positions unchanged. The HOT system is built on an inverted Nikon microscope (Eclipse TI, Nikon, Tokyo, Japan) with a continuous-wave fiber laser (*λ* = 1070 nm, THFL-1P400-COL50, BKtel Photonics, Lannion, France) as a light source, an Olympus water immersion objective (UPlanSApo 60X, NA = 1.2) as a focusing element, a motorized microscope stage (ProScan, Prior Scientific, Fulbourn, UK) for sample translation and a spatial light modulator (PLUTO NIR, Holoeye, Berlin, Germany) to generate the multiple traps. The total optical power at the entrance of the objective pupil was 270 mW, which, considering the approximately 50% transmittance of the objective at 1070 nm, resulted in ~34 mW power for each trapping beam. The sample was observed with an EMCCD camera (Rolera EMC2, Qimaging, Surrey, BC, Canada).

### 2.4. Cell Indentation Experiment

The experimental arrangement is depicted in Figure 1c,d. Figure 1c shows the cells grown on the vertical wall polymerized onto a glass substrate, and the microtool approaching the cells in the direction which is perpendicular to the wall surface, to the cells surface and to the optical axis of the trapping objective, and parallel to the supporting glass surface. Figure 1d illustrates the sample assembly and microtool alignment procedure with the cells already present on the SU8 walls. First, the microtools, which were collected from their polymerization glass support, were pipetted into the well containing the cells (see Section 2.2). In the well, the cells were immersed in about 200–300 μL of Leibovitz’s L-15 medium (Sigma) that kept them vital without CO_2_ incubation for the approximately 2-h duration of the experiments. At this stage, the structures were scattered randomly on the bottom of the sample well.

**Figure 2:**
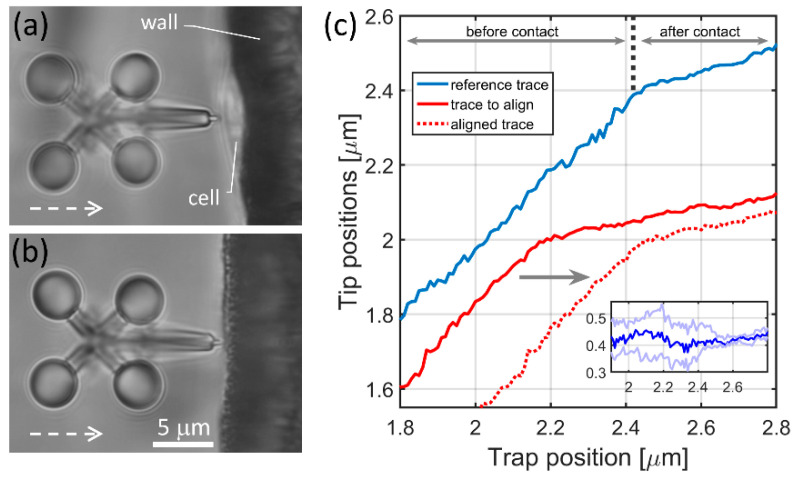
Cell indentation experiments and the resulted traces of the microtool’s tip. Panels (**a**,**b**) show a typical snapshot of the cell indentation and the wall approaching experiments, respectively; the optical axis is perpendicular to the plane of the figure, the white dashed arrows indicate the direction of the microtool movement during the indentation experiment. The tip position was calculated by determining the positions of the four handle spheres on the image series taken during the indentation experiments. (**c**) shows tip positions from two cell indentation experiments as the function of the trapping beam position (solid blue and solid red traces). It also shows the result of the trace alignment procedure when the red trace is aligned to the blue one with the alignment procedure (dashed red). The inset in (**c**) shows differences of the red and blue traces during the alignment procedure (see main text).

For the measurement of the Young’s modulus, the force that pushes the microtool to the target cell and the cell indentation need to be determined. For both values, the tool’s position needs to be measured precisely. The force is calculated from the displacement of the microtool relative to the trapping foci. The indentation was determined relative to the case when it is pushed against a hard wall instead of a soft cell. The difference in the movements in these two cases provided the value of indentation, as described below. In both cases, the microtool was translated in a well-controlled manner: after taking hold of it with the optical trap, it was elevated from the substrate to about 5–15 μm above it by defocusing the objective and aligning it with the plane of its four spheres perpendicular to the optical axis. When elevated, the trap stayed fixed relative to the trapping objective and the sample stage was moved until a proper target cell arrived in the field of view (sample movement is shown by the blue dashed arrows on Figure 1d Step 2). Then, with a stationary sample stage, the microtool was rotated towards the target cell by moving the trapping focal spots, until the microtool’s tip aligned with the normal of the wall’s surface (with or without the cells on it) (Figure 1d Step 3 and Figure 2a,b). After the microtool was oriented towards the cell, selected upon visual inspection, the cell’s silhouette was brought into focus together with the tool’s tip by the consecutive adjustments of the focusing objective and the trap position along the optical axis; this ensured that the point of attack on the cell was seen as a sharp contour. Then, the microtool approached the wall to about 2 μm, moving only the microscope stage (rough approach), and stopped. In this moment, the tool was situated at about 5–15 μm height from the supporting glass, which ensured that the trapping foci were only minimally, or not at all distorted by the bottom area of the cell-supporting wall. In step 4, the indentation experiment commenced, when the optical trap was moved in 10 nm steps towards the wall, using only the HOT (fine approach), and at each position a bright-field image of the microtool was recorded; this process resulted in an average speed of 0.05 μm/s. The trajectory of the tip of the microtool was parallel to the normal of the wall. Before making contact with the wall or the cell, the position of the microtool and the trap coincided. When the microtool reached the wall, the movement of the trapping foci continued as before for about an extra 1 μm, but the tool was retarded relative to the trap position. This retardation provides a force by which the microtool is pushed against the wall or the cell surface. Finally, the microtool was retracted and positioned to the next available cell. When more than one indentation experiment was carried out on the same cell, we probed the cell at points that were a few hundreds of nanometers away from each other. Altogether, 19 measurements were carried out on 6 cells with 4 manipulators.

### 2.5. Data Analysis

The position of each microtool during the approach of the cell was determined with a correlation-based method where the reference was its image at the very first position. The script was implemented in Matlab, using built-in image processing and 2D cross-correlation functions. The positions of the four trapping spheres were determined independently. First, a template image was chosen, which was the cropped image of the selected sphere on the very first frame. Then, this reference image was compared to all consecutive frames using Matlab’s built-in *normxcorr2* function. This function resulted in a correlation matrix for each frame with the same size as the frame itself. The maximum of this matrix provides the position on each frame where its similarity to the reference image is the largest; in other words, this maximum is the new position of the sphere on the frame. One must pay attention to the fact that *normxcorr2* provides this position only to one pixel size precision, which was 120 nm in our case. In order to find the position with sub-pixel precision, the values around the correlation matrix maximum value were fitted with a 2D Gaussian function, and the center of this Gaussian gave the new location of the sphere with sub-pixel precision. Next, the position of the microtool’s tip was calculated from these sphere position data taking advantage of the fact that the tool is a rigid structure and that it moves in the focal plane. The reason why the image of the tip itself was not monitored is that after it makes a direct contact with the cell, the image becomes distorted and it cannot be used for cross-correlation. At the end of this process, the position of the tip was determined for all the frames and could be plotted as the function of the trap position, which changes between frames by 10 nm.

The precision of the correlation-based position determination method was found to be 5.5 nm FWHM as measured on surface-attached, non-moving microtools. For this, 2000 frames of the surface-attached microtool was recorded and analyzed with the correlation-based method; in theory, the measured positions of the four spheres should not change between frames. In reality, small fluctuations were measured partly due to residual mechanical vibrations and to the imprecision of the calculation of the correlation. The positions of the tip were determined along as well as perpendicular to the direction of the optical trap movement; only those attempts were eventually used in the analysis where the tip movement perpendicular to this direction is negligible (smaller than 50 nm) after the contact.

The result of the image processing is a microtool tip position vs. trapping focus position trace for each indentation experiment. These traces have two distinct ranges as shown in Figure 2c: the first one describes the movement before the contact the microtool makes with its target; in this range, the microtool follows the trap position precisely, so its slope is 1 (reference trace before 2.42 μm in Figure 2c). After the microtool makes contact with its target (a cell, a wall or a bead, see below), it lags behind the trap, so the slope of this range becomes less than 1 (reference trace after 2.42 μm in Figure 2c). Since the contact point did not fall to the same trap position in the consecutive experiments, the traces needed to be aligned. The cell indentation experiment series and the wall approach experiments resulted in two distinct sets of traces. The cell indentation traces were aligned to each other with one alignment procedure, so were the wall approach traces with a separate procedure. In each procedure, a reference trace was selected from the experiments (usually the first one), and the rest of the traces were aligned to it. The alignment was based on calculating the variance of the difference of two traces while one of them (red curve in Figure 2c) was shifted stepwise in respect to the other one that served as a reference (blue curve in Figure 2c) (the step size was 10 nm). The minimum of the calculated variances gave the amount of trace shift used for alignment. The inset in Figure 2c shows three of such difference traces: the dark blue is the case of minimum variance, while the other two have a variance 3 times larger. After aligning the cell and wall approach experiments, the traces from the wall experiments were averaged (*n* = 9), while those of the cell experiments were used individually later to calculate cell indentation and the displacement of the microtool.

### 2.6. Trap Stiffness Calibration

**Figure 3:**
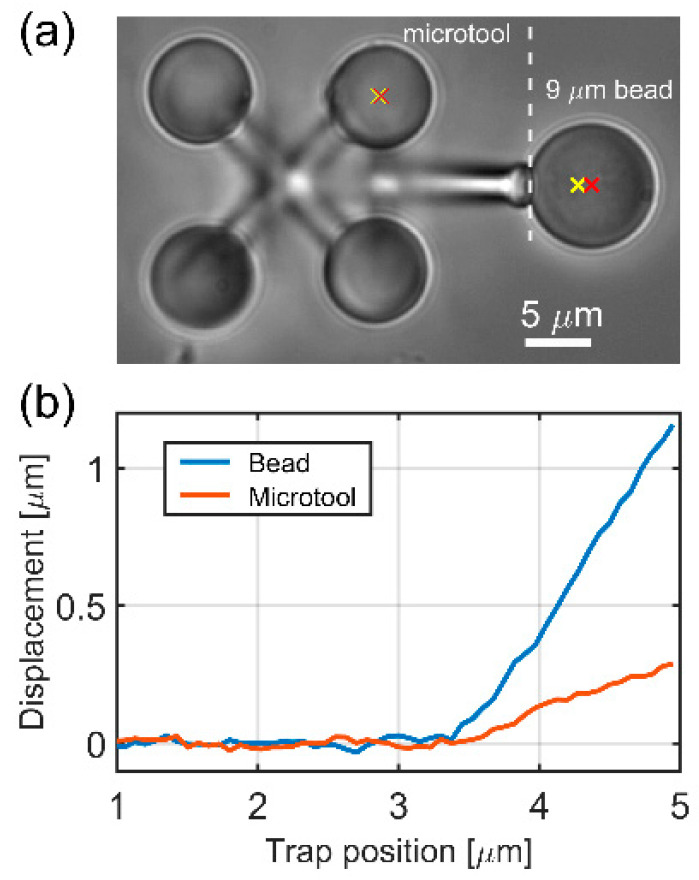
Trap stiffness calibration for the cell indenter microtool. Panel (**a**) shows the optical microscopic image of the tool (**left**) and the 9 μm bead (**right**) during the calibration experiment. The yellow crosses show the positions of two optical traps, one holding one of the spheroids of the microtool, the other holding the 9 μm bead. The red crosses show the center of one of the spheroids on the microtool and that of the bead. The distance of the yellow and red crosses gives the displacement values plotted on (**b**).

For the calculation of the Young’s modulus, the force that the microtool exerted on the cell creating the measured indentation must be known. This force is calculated as the displacement of the microtool from the equilibrium position multiplied by the trap stiffness. The microtool’s trap stiffness ($k_{m}$) was measured with an indirect method. Here, the microtool was pushed against a trapped 9 μm polystyrene bead of known trap stiffness ($k_{b}$) and the displacement of the microtool was compared to that of the bead (Figure 3). First, $k_{b}$ was determined by trapping the bead alone using the equation

(1) $$\frac{1}{2}k_{B}T\;=\;\frac{1}{2}k_{b}\langle x^{2}\rangle \;$$

where $\langle x^{2}\rangle$ is the variance of the bead fluctuation determined by video tracking (using 0.5 ms exposure time), *T* is room temperature (295K) and $k_{B}$ is Boltzmann’s constant [29]. Then, the microtool was pushed against the trapped bead and the following equation resulted in the trap stiffness of the microtool:

(2) $$k_{m}=k_{b}\times \frac{\Delta x_{b}}{\Delta x_{m}}\;$$

where $\Delta x_{b}$ and $\Delta x_{m}$ are the displacements of the bead and the microtool, respectively. For this measurement, the tip of the microtool was slightly modified: instead of a sharp tip, it had a flat one; this modification was micrometers away from the trapped spheroids of the tool, so it did not affect the trap stiffness.

## 3. Results

An example for the polymerized microtools we used for the cell indentation experiments is shown in Figure 1. The four spheres used to hold the tool with the optical tweezers have 6.5 μm diameter and their centers form a 14 μm × 14 μm square. The apex of its tip has an ellipsoid shape with the smallest radius of 100 nm and a large one of 500 nm; this shape is inherited from the inherent shape of the basic building block of TPP. In our calculations, we take an average radius of 300 nm for the microtool tip. For the trap stiffness measurement, the tip was modified to a 2 μm × 2 μm flat end.

### 3.1. Trap Stiffness Calibration

In Figure 3a, the tip-modified microtool and a 9 μm bead are shown during the stiffness measurement. When the microtool is pushed against the bead in 50 nm steps, both the microtool and the bead are displaced from their equilibrium positions. Since the force that the microtool exerts on the bead and that the bead exerts on the microtool is equal, Equation (2) can be used to calculate $k_{m}$. Equation (1) resulted in a $k_{b}$ bead stiffness of 4.5 pN/μm. Figure 3b depicts the averaged bead and microtool displacements as the function of the trap position. From these curves, $k_{m}$ can be calculated for a range that starts about 0.5 μm after the contact point (between 4 and 5 μm); the obtained microtool stiffness value is 16.49 ± 2 pN/μm.

### 3.2. Endothelial Cells Young’s Modulus

**Figure 4:**
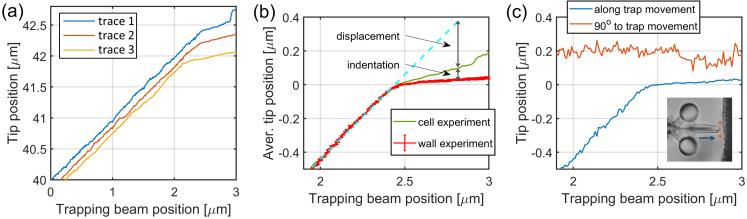
Tip position traces for the calculation of endothelial cell’s Young’s modulus. Panel (**a**) shows representative raw tip position traces as the function of the trap positions, before alignment, obtained from individual cell indentation experiments. After the alignment procedure, the tip positions (**b**) of the wall approach experiments were averaged for background (red curve), while the cell indentation traces (an example is shown by the green curve) were used individually to calculate indentation and displacement. The error bars on the red trace represent standard deviation. At each trapping beam position, the cell indentation was calculated as the difference between the green and red curves, and the microtool displacement as the difference between the green curve and the trapping beam position (dashed light blue curve). Panel (**c**) shows a tip position movement parallel (blue) and perpendicular (red) to the trap movement (that is, axis of the microtool) during the tool being pushed against a hard wall. The inset displays the tip movement along the trap progression (blue line) and perpendicular to it (red line).

The Young’s modulus was calculated according to the equation used in the literature for indentation experiments performed with AFM or optical tweezers (Hertz model):

(3) $$F\left(d_{z}\right)=\frac{4E}{3\left(1-\nu^{2}\right)}R_{b}^{1/2}d_{z}^{3/2},$$

where *F* is the force at which the indenter is pushed against the cell, *E* is the Young’s modulus, *R_b_* is the radius of the indenter surface, *d_z_* is the indentation and *v* is the Poisson number (we chose 0.5) [21,22]. The indentations and the forces (in the form of displacement) were calculated from the microtool position traces as the function of the trap positions. Figure 4a shows representative raw microtool tip positions during the cell indentation experiments before their alignment. The individual traces illustrate that the microtools’ movement changes radically after they made contact with the cells: they do not follow the movement of the trap but do not stop completely either. After the contact, the movement continues to be primarily a linear function of the trap position for at least another 500–800 nm of trap movement; in this regime, the tip moves less than 150 nm. In a few cases, the tip position suddenly increased after about 100 nm tip travel due to an occasional sideway slip on the cell membrane. Figure 4b shows one aligned tip position trace when pushed against a cell (green curve) and the average of the traces from the approaches of the hard SU8 walls (red curve). The tip position is meaningful mainly in the first 400 nm beyond the contact point, where only negligible slipping took place. In the case of the hard wall, the tips usually do not stop completely but a residual forward movement remains, which is due to the small sideway movements of the tip on the surface. We believe that these small slips also took place for the cell experiments, so the extra average forward movement observed at the walls was used as a “baseline” in the cell indentation experiments: the tip positions from the cell experiments were compared to this baseline. Figure 4c shows a typical experimental result of an approach of the hard wall. It is visible that the tip continued to move forward about 20 nm during the first 400 nm of trap position movement after the contact (between 2.43 and 2.83 μm), while it slipped sideways at an average of 50 nm.

**Figure 5:**
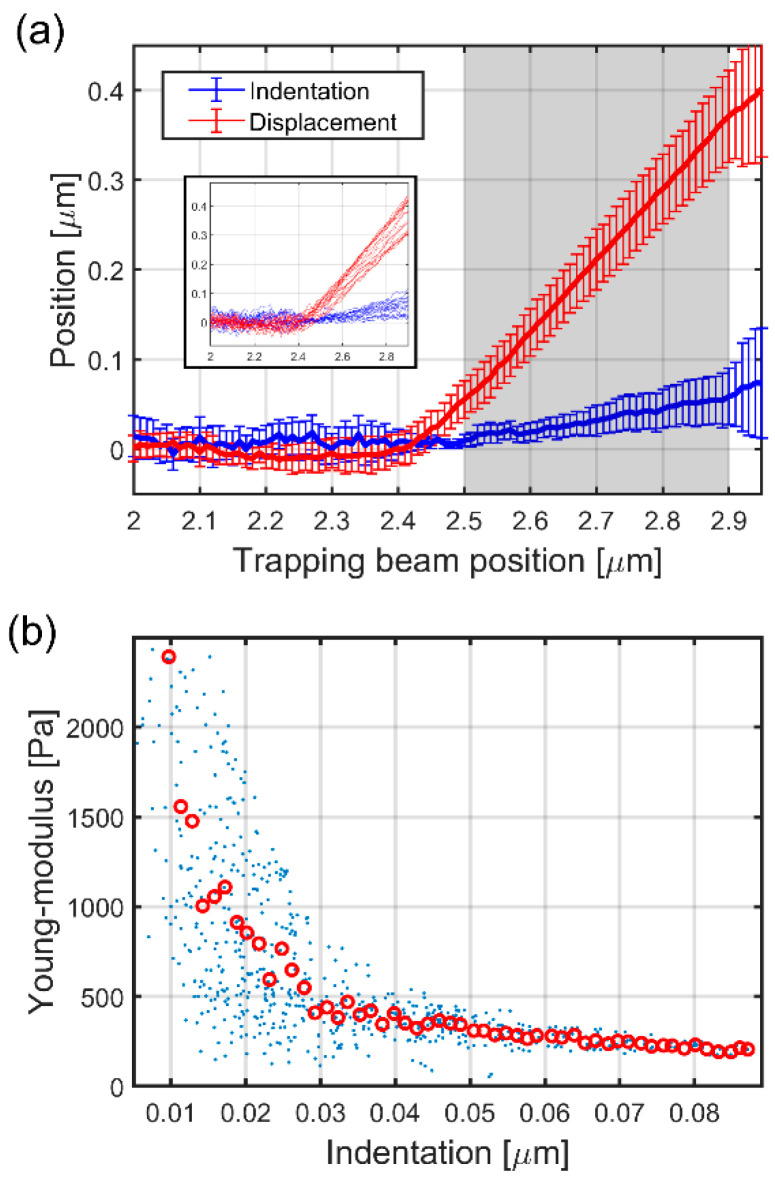
The measured indentation and displacement data and the Young’s modulus calculated from them. The indentation (blue line) and displacement (red line) data on panel (**a**) are calculated from the aligned traces of the 19 cell indentation experiments as shown in Figure 4b; the error bars represent standard deviation. The shaded area highlights the reliable range for the two quantities. The inset shows the individual displacement traces (red) and indentation traces (blue) calculated separately for the 19 experiments. The Young’s modulus as the function of indentation over the values highlighted in (**a**) is shown in panel (**b**). The blue dots represent all of the approximately 800 individual point pairs (40 trap positions × 19 experiments), while the red circles are their averages in 60 regions over the 0–0.09 μm indentation range.

The two position traces illustrated in Figure 4b were used to calculate the indentation and force values used in the Hertz model for each individual cell indentation experiment. The indentation is simply the difference between the tip positions when approaching the cell and when approaching the wall. The displacement was calculated by first fitting a straight line to the initial part of the cell approach trace (light blue dashed line in Figure 4b) and then taking the difference between this line and the tip position after the contact point. Figure 5a shows these two values, the cell indentation and the microtool displacement as the function of the trap position. The applied force is calculated from the displacement by multiplying it by the trap stiffness $k_{m}$. The displacement resulted in a force ranging from 1 to 5 pN, which is below the precision of an AFM. Both traces have a break at the contact point between 2.4 and 2.5 μm, but produce a large error of about 400 nm after the contact point. The displacement and indentation values can be measured reliably in the trap position range of 2.5–2.9 μm, therefore the Young’s modulus can be regarded as reliable also only here. We obtained values ranging from about 220 up to about 1500 Pa (Figure 5b), although between the 2.5 and 2.6 μm trap positions (corresponding indentation: between 0.01 and 0.02 μm), the values could be determined with significant noise.

## 4. Discussion

We have designed an optical tweezers-operated microtool specifically to measure the elasticity of adherent cells in closed microfluidic environments. In contrast to earlier optical trap-based cell indentation experiments, this tool approaches the target cells laterally which makes the determination of its position easier even with simple bright-field video microscopy. In addition, in the trapped parts of the structures, the four spheroids are more than ten micrometers away from the probe tip, which ensures that the optical field does not cause photodamage to the cells. The analysis of the microtools’ position during the indentation experiments revealed that they move in a straight and continuous way after making contact with the cell, and their position values yield the indentation and pushing force values in a straightforward manner. The sideways slip of the microtools’ tip in contact with the cells could be readily detected and excluded from the evaluation based on its magnitude. Residual sideways tip movements could be compensated for with a control measurement using a hard wall without cells. The microtools were characterized with 16.5 pN/μm trap stiffness and pushing force in the 1–5 pN range, comparable to other optical trap-based elasticity measurements but well below that of an AFM. This force together with the 300 nm tip radius of the microtool yielded the measured indentation values of up to 90 nm. The operating force range can be easily extended to higher values with higher trapping laser power and smaller trapping sphere diameters.

We obtained Young’s moduli in the range between 220 and 1500 Pa, depending upon the position of the trapping beam, consequently on the amount of indentation; the noise of the Young’s modulus, however, remarkably increases with the decrease in the indentation. These values are comparable to those measured by AFM on bovine aortic endothelial cells (700–2.7 kPa [30]), on pulmonary artery endothelial cells (400–1500 Pa [31]), on human umbilical vein endothelial cells (HUVEC) (350–4000 Pa [21]) or by magnetic tweezers on HUVEC (400 Pa [32]). It is noticeable that the values for the AFM-derived Young’s modulus can easily vary an order of magnitude across the literature for the same type of cell. For endothelial cells, values anywhere from ~200 to ~5000 Pa can be found; our measured Young’s modulus falls to the lower regime with a few hundred Pa. The main reasons for this broad range can be found in the measurement conditions: mainly in the rate and amount of indentation, and in the shape of the object the indentation is realized with.

It was shown that increasing the indentation rate increases the apparent Young’s modulus due primarily to viscous effects [20,33]. The typical loading rates used in an AFM measurement span from 100 pN/s [20] to tens [33] or hundreds of nN/s values (exerting 1 nN force with 0.5 kHz frequency of the AFM cantilever [34]). Our optical tweezers approach experiment lasts about 60 s, where during only the last 5–6 s does the tip actually hit the cell. Considering the averaged maximum of 60 nm indentation and that by the end of this period the force increases to an average of 6 pN, it yields an indentation rate as low as 0.01 μm/s and a loading rate of about 1 pN/s, which is orders of magnitude smaller than those of AFM. For Mathur and co-workers, the lower limit for viscous dissipation was at 0.25 μm/s probe velocity [33]. We are confident that at the observed low-indentation rate viscous effects do not play any role in measuring the Young’s modulus in our experiments.

In the papers of Vargas-Pinto and Mathur [21,33], the authors also showed that the higher the indentation, the lower the Young’s modulus, similarly to our results (Figure 5b). The amount of indentation, which for us was up to 90 nm, is in the lower regime of what was obtained with AFM or optical tweezers [21,24]; it is very likely that with this low value, we mainly measure the elastic properties of the cell membrane and not the complex characteristics of the underlying actin network. The measurement error of Young’s modulus increases significantly below 30 nm indentation. It is believed that this noise is primarily due to thermal fluctuations and the increase in the relative error when using small indentation and force values in Equation (3). However, the larger *E* for lower indentations is elsewhere argued to originate from the nonlinear elasticity of the cell [33]; it is beyond the scope of this paper to study the nonlinear phenomenon in detail. The third important parameter is the shape of the intender. Vargas-Pinto et al. reported on using AFM tips with radii of curvature from 20 nm to 5 μm for endothelial cell indentation [21]. They found that while the sharp tip yielded a value of 3.8 kPa for the Young’s modulus, the 5 μm one yielded only 350 Pa for the same type of cell. Similarly, Harris and co-workers found that pyramidal sharp tips can measure double the Young’s modulus of that measured with spherical (*r* = 7.5 μm) tips (800 vs. 400 Pa) on MDCK cells [35]. Chiou and co-workers also observed a more than two-fold increase in the Young’s modulus value for mouse fibroblast cells when they compared sharp pyramidal tips with flat top (diameter of 1.8 μm) and spherical (sphere *r* = 2.5 μm) tips [36]. The tip of our microtool has a radius of 300 nm on average, which is much larger than that of the conical AFM (*r* ≈ 10 nm) tips and comparable to those used for optical tweezers indentation (*r* = 0.4–1.5 μm); this size also points towards measuring Young’s moduli in the lower few hundreds of the Pa regime with our microtool.

In conclusion, the measurement of a cell’s Young’s modulus requires a careful approach in order to obtain reliable results. Even with one technique, one can measure very different values depending on the measurement parameters. The solution probably lies in what one actually wants to measure. If one is interested in the pure linear elastic properties of the cell, it is believed that the use of large radius of curvature indentation surfaces, small indentations (with small forces) and small loading rates is more appropriate to characterize specifically that. Optically micro-manipulated polymer structures should ideally operate in this regime.
